# Real-life observational study on niraparib in older patients with primary tubo-ovarian cancer: a focus on safety and efficacy

**DOI:** 10.1007/s10147-025-02914-y

**Published:** 2025-11-04

**Authors:** Adriana Ionelia Apostol, Matteo Bruno, Carolina Maria Sassu, Serena Maria Boccia, Laura Vertechy, Giorgia Russo, Ilary Ruscito, Filippo Maria Capomacchia, Giovanni Scambia, Anna Fagotti, Claudia Marchetti

**Affiliations:** 1 Dipartimento Scienze Della Salute Della Donna, del Bambino E Di Sanità Pubblica, Fondazione Policlinico Universitario Agostino Gemelli, IRCCS, Via Largo Francesco Vito 1, Rome, Italy; 2 Dipartimento Scienze Della Vita E Sanità Pubblica, Università Cattolica del Sacro Cuore, Rome, Italy

**Keywords:** Niraparib, Elderly, Ovarian cancer, Maintenance

## Abstract

**Background:**

Niraparib is approved for maintenance treatment of tubo-ovarian cancer patients, but data on older patients are limited. This retrospective study evaluated its safety and efficacy in primary advanced tubo-ovarian cancer, focusing on patients ≥ 75 years.

**Methods:**

Women aged ≥ 50 years diagnosed with primary high-grade serous tubo-ovarian cancer, treated with niraparib between 2019–2023, were enrolled. Patients were stratified into three groups: A (50–64 years), B (65–74 years), and C (≥ 75 years). The primary outcome was progression-free survival. The secondary outcomes were toxicity and dose reduction.

**Results:**

127 patients were identified: 62 (48.8%) group A, 26 (20.5%) group B, and 39 (30.7%) group C. Baseline characteristics were comparable across groups, excluding a higher proportion of interval cytoreductive surgeries (*p* = 0.001), residual tumor (*p* = 0.01) and Eastern Cooperative Oncology Group (ECOG) > 1 (*p* = 0.01) in group C. Most patients started niraparib at 200 mg/day with dose reductions primarily occurred within fourth cycle. Dose reductions were observed in 77.4%, 69.2% and 56.4% of patients in groups A, B, and C, respectively (*p* = 0.08). In patients ≥ 75 years, 26 (66.7%) discontinued treatment due to disease progression (48.7%) or toxicity (17.9%). There were no significant differences in common or grade ≥ 3 adverse events between groups. Progression-free survival was 12 months (95%CI: 2.0–25.0) for group A, 29 months (95%CI: 11.0–52.0) for group B, and 16 months (95%CI: 1.0–31.0) for group C (*p* = 0.78).

**Conclusions:**

Our findings suggest that niraparib is safe and well-tolerated in aged ≥ 75 years. Concerns about toxicity should not preclude the enrollment of elderly patients in treatment regiments.

**Supplementary Information:**

The online version contains supplementary material available at 10.1007/s10147-025-02914-y.

## Introduction

Approximately 22% of newly diagnosed tubo-ovarian cancer patients are over the age of 75 and most of them present with advanced-stage disease (International Federation of Gynecology and Obstetrics (FIGO) stage III-IV), requiring an approach that combines surgery, chemotherapy and maintenance therapy as critical components of care [1]. Even though the older population (≥ 75 years) represents a significant percentage, limited data are available on toxicity and its management.

Older patients present different characteristics than their younger counterparts; factors such as comorbidities, performance status, and geriatric complexities create additional challenges for clinicians in tailoring treatments. Notably, the elderly population is often underrepresented in various randomized clinical trials resulting in a lack of data to manage the use of Poly (ADP-Ribose) Polymerase (PARP) inhibitors in real-world settings [2–5]. This complex clinical management is also due to the possible pharmacological interactions between PARP inhibitors and drugs usually taken by these patients: approximately 78% of population over 70 years receives at least one drug, and about 39% receive three or more [6, 7].

In the retrospective analysis of the ENGOT-OV16/NOVA trial, older patients ≥ 70 years receiving niraparib as a maintenance therapy exhibited adverse event profiles comparable to younger cohort [8]. Moreover, subgroup analyses demonstrated that niraparib significantly provided an improvement in progression-free survival for patients aged 65 and older [9]. These findings suggest that older age does not impact the therapeutic efficacy of niraparib, although a tailored management is required to mitigate toxicity. Similar data about niraparib in the real-world setting are currently lacking, therefore we conducted this real-life study to investigate the safety and efficacy of niraparib in maintenance therapy in primary advanced tubo-ovarian cancer focusing on patients ≥ 75 years. We aimed to ultimately provide information on the correct management in this elderly group enrolled in a setting of single-center retrospective study.

## Materials and methods

This is a retrospective, single-center, cohort study. The Institutional Review Board of Policlinico Universitario Agostino Gemelli, IRCCS approved the study (ID: 6356). Inclusion criteria were histologically confirmed primary high-grade serous ovarian cancer or fallopian tube carcinoma; FIGO stage III-IV, patients aged ≥ 50 years treated with niraparib between 2019 and 2023 were included. Exclusion criteria were recurrent tubo-ovarian cancer, use of PARP inhibitors as part of an experimental protocol, patients who started niraparib less than 6 months at the time of data analysis and who continued therapy at another center; age < 50 years. To gain a more comprehensive understanding of outcomes in elderly population (≥ 75 years), we stratified them into three groups: group A (50–64 years), group B (65–74 years), and group C (≥ 75 years). The starting dose of niraparib was an individualized starting dose based on weight < or ≥ 77 kg, platelet count < or ≥ 150,000/µL, according to Berek et al. [10]*.* In patients aged 75 years or older, additional considerations for initial dosage were guided by the ECOG (Eastern Cooperative Oncology Group) status, comorbidities and residual toxicity from previous platinum-based chemotherapy. Maintenance treatments in patients aged ≥ 75 years are summarized in Supplementary Fig. 1.

All patients underwent regular monitoring with visits scheduled every 28 days to document toxicities according to the National Cancer Institute Common Terminology Criteria for Adverse Events v5.0 [11]. Hematological toxicity was closely monitored during the first eight weeks, with weekly blood counts performed. Subsequently, biweekly blood tests were performed, except in cases requiring closer monitoring due to hematologic effects. CT or PET-CT scans was required to evaluate disease progression. The primary outcome was progression-free survival, defined as the time elapsed between the start of treatment with PARP inhibitors and documentation of progressive disease or the date last seen. Secondary outcomes included rates of toxicity and dose reduction.

The whole data are summarized by descriptive statistics measures. Categorical variables were compared using the chi-square test or Fisher’s exact test, as appropriate. Continuous variables were analyzed using the Kruskall-Wallis test or Mann–Whitney U test. Progression-free survival was estimated using Kaplan–Meier method and compared using the Cox proportional hazards model. The log-rank test was applied to assess statistical significance between survival curves. Logistic regression was used to assess predictors of dose reduction, with results expressed as odds ratios (OR) and 95% confidence intervals (CI). All statistical tests were performed using the Statistical product and Service Solutions software (SPSS, version 29.0; BM Corp., Armonk, NY, United States). Statistical tests were two-sided, and differences were considered significant at the level of *p*-value < 0.05.

Propensity score matching was performed through R Studio software version 2024.12.1 + 563 (Posit Software, PBC). Patients aged < 75 years and those aged ≥ 75 years were matched according to the following variables: *BRCA* mutational status, type of surgery (primary debulking surgery versus interval debulking surgery), FIGO disease stage, and residual tumor after cytoreductive surgery.

## Results

### Characteristics of study population

A total of 127 patients who received first-line maintenance therapy with niraparib were included: 62 patients (48.8%) in group A, 26 patients (20.5%) in group B, and 39 (30.7%) in group C. The baseline characteristics of the patients are summarized in Table 1. A different surgical approach was found to be relevant between three groups (*p* = 0.001); indeed, group C had a lower number of patients who underwent primary debulking surgery compared to group A. Additionally, in the older cohort, it was noted that 9 patients (23.1%) did not undergo surgical cytoreduction, which contrasts with the younger patients. This difference is also reflected in the varying rates of optimal cytoreduction achieved across the groups as well as in the distribution of residual disease observed in Group C (*p* = 0.01). Moreover, older patients exhibited more significant comorbidities and higher frequency of Eastern Cooperative Oncology Group (ECOG) 1 compared to younger counterparts (*p* = 0.01). Differently, baseline hematologic profiles were similar in all three groups (*p* > 0.09). All patients received individualized starting dose: the most common niraparib maintenance starting dose in all groups was 200 mg/day. However, the distribution of starting doses significantly differed among age groups (*p* = 0.003), with patients aged ≥ 75 years more frequently receiving a lower starting dose (Table 2). In group C, 9 patients received a maintenance starting dose of 100 mg/day: all these patients had an ECOG 1 with residual toxicity from previous chemotherapy (fatigue, anemia, neutropenia), and four of these patients had major cardiovascular diseases.

**Table 1:** Baseline characteristics of patients

Variables	Group A 50–64 y n = 62	Group B 65–74 y n = 26	Group C ≥ 75 y n = 39	*p*-value
**Median weight at niraparib starting dose**, kg (range)	58 (40–92)	58 (40–82)	60 (46–80)	0.80
**FIGO stage at diagnosis, n (%)**				
IIIA-IIIC	43 (69.4%)	16 (61.5%)	24 (61.5%)	0.65
IVA -IVB	19 (30.6%)	10 (38.5%)	15 (38.5%)	
***BRCA*** **status, n (%)**				
* BRCA1* mutated	0	1 (3.8%)	1 (2.6%)	0.35
* BRCA2* mutated	0	0	0	
* BRCA* wild-type	62 (100%)	25 (96.2%)	38 (97.4%)	
**HRD status, n (%)**				
HR deficient	8 (12.9%)	2 (7.7%)	4 (10.3%)	0.55
HR proficient	10 (16.1%)	6 (23.1%)	4 (10.3%)	
Unknown^a^	44 (71.0%)	18 (69.2%)	31 (79.5%)	
**Surgery, n (%)**				
Primary cytoreductive surgery	34 (54.8%)	9 (34.6%)	12 (30.8%)	**0.001**
Interval cytoreductive surgery	28 (45.2%)	17 (65.4%)	18 (46.2%)	
No cytoreductive surgery	0	0	9 (23.1%)	
**Residual tumor, n (%)**				
Residual tumor = 0	58 (93.5%)	21 (80.8%)	26 (66.7%)	**0.01**
Residual tumor ≤ 1 cm	4 (6.5%)	5 (19.2%)	1 (2.6%)	
Residual tumor > 1 cm	0	0	3 (7.7%)	
No cytoreductive surgery	0	0	9 (23.1%)	
**Number of chemotherapy cycles, n (%)**				
≤ 6	61 (98.4%)	23 (88.5%)	37 (94.9%)	0.13
> 6	1 (1.6%)	3 (11.5%)	2 (5.1%)	
**ECOG, n (%)**				
0	60 (96.8%)	21 (80.8%)	17 (43.6%)	**0.01**
1	2 (3.2%)	5 (19.2%)	22 (56.4%)	
**Time to start niraparib**				
< 40 days	4 (6.5%)	3 (11.5%)	5 (12.8%)	0.52
> 40 days	58 (93.5%)	23 (88.5%)	34 (87.2%)	
**Median platelets level at starting niraparib** (range, 10^3^/μL)	238 (157–597)	228 (125–572)	239 (132–620)	0.88
**Median haemoglobin level at starting niraparib** (range, g/dl)	12.1 (10–14.5)	12.5 (9.8–13.8)	11.8 (9.4–13.5)	0.71
**Median neutrophils level at starting niraparib **(range, 10^3^/μL)	2.37 (1.4–5.9)	3.15 (1.27–6.79)	2.9 (1.02–4.9)	0.09

Bold highlights statistically significant *p*-values

*FIGO* International Federation of Gynecology and Obstetrics, *HRD* Homologous Recombination Deficiency, *HR* homologous recombination, *ECOG* Eastern Cooperative Oncology Group

^a^HRD status was available for few patients due to the time interval considered (2019–2023)

**Table 2:** Treatment exposure, safety, and adverse events stratified by age groups

Variables	Group A 50–64 y n = 62	Group B 65–74 y n = 26	Group C ≥ 75 y n = 39	*p*-value
**Median treatment exposure** (days)	335	333	222	n/a
**Niraparib starting dose, n (%)**				
300 mg/day	3 (4.8%)	0	0	**0.003**
200 mg/day	59 (95.2%)	25 (96.2%)	30 (76.9%)	
100 mg/day	0	1 (3.8%)	9 (23.1%)	
**Total number of Treatment-Emergent Adverse Events (%)**	62 (100%)	26 (100%)	39 (100%)	1.00
**Any grade ≥ 3 Treatment-Emergent Adverse Events (n, %)**				
Anaemia	17 (28.3%)	1 (3.8%)	5 (12.8%)	
Neutropenia	12 (19.4%)	0	6 (15.4%)	
Thrombocytopenia	15 (24.2%)	4 (15.4%)	13 (33.3%)	
Hypercreatinemia	0	0	0	
Fatigue	2 (3.2%)	4 (15.4%)	1 (2.6%)	
Gastrointestinal disorders	0	0	0	
Other disorders	0	2 (7.7%)	3 (7.7%)	
**Any Treatment-Emergent Adverse Events leading to dose reduction, n (%)**	48 (77.4%)	18 (69.2%)	22 (56.4%)	0.08
**Any Treatment-Emergent Adverse Events leading to dose interruption, n (%)**	4 (6.5%)	5 (19.2%)	7 (17.9%)	0.12

Bold highlights statistically significant *p*-values

*n/a* not applicable

### Oncological outcomes

**Fig. 1:**
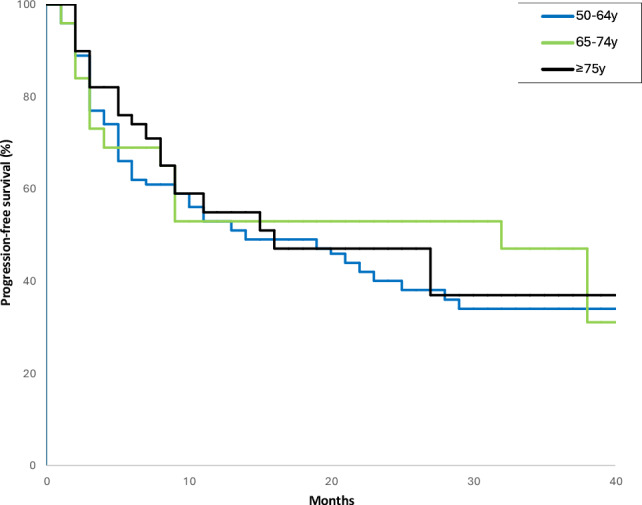
Progression-free survival across the three groups: **a** (50–64 years), **b** (65–74 years), and **c** (≥ 75 years)

**Fig. 2:**
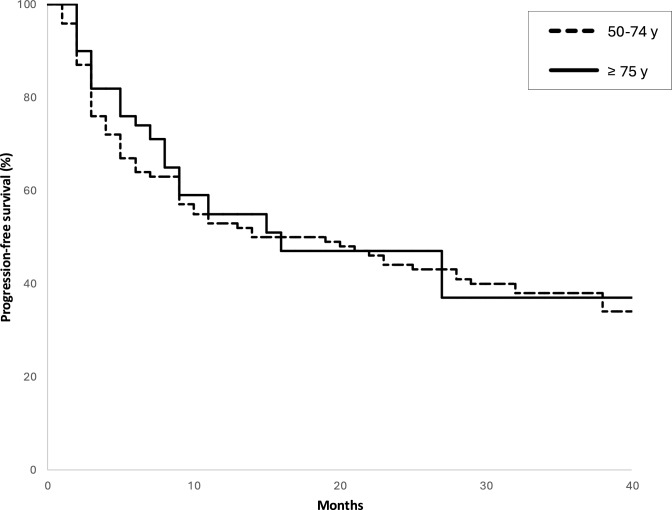
Progression-free survival in patients 50–74 years (groups A + B) versus ≥ 75 years (group C)

As of December 2024, the median follow-up for all groups was 32.8 months (range: 11.0–46.0). Relapse after maintenance therapy was documented in 39 (37.1%) patients in group A, 14 (46.2%) in group B and 19 (51.3%) in group C. From the analysis of the three main groups, progression-free survival was not significantly affected by dose reduction over the treatment. Indeed, progression-free survival was evaluated across the groups: 12 months (95%CI: 2.0–25.0) for group A, 29 months (95%CI: 11.0–52.0) for B, and 16 months (95%CI: 1.0–31.0) for group C. The Kaplan–Meier analysis showed no significant differences in progression-free survival between groups with hazard ratio (HR) of 0.93 (95%CI: 0.47–1.98; *p* = 0.78) (Fig. 1). Moreover, considering the different number of patients who did not underwent cytoreduction (23.1% of group C versus 0% in group A and B; Table 1); we additionally performed survival curves among the three groups, excluding the patients in group C who never underwent cytoreduction: no significant difference was observed among the three populations in terms of progression-free survival (HR 0.86, 95%CI: 0.45–2.41; *p* = 0.37) (Supplementary Fig. 2). Finally, even combining groups A and B and comparing their prognosis with group C, the progression-free survival remains comparable, with no statistically significant difference observed (*p* = 0.78) (Fig. 2).

### Propensity score matching analysis

Propensity score matching was performed, selecting 39 patients per age group (< 75 years and ≥ 75 years). Baseline characteristics are reported in Supplementary Table 1**.**

No statistically significant differences in progression-free survival were observed between the two age groups (*p* = 0.66), as shown in Supplementary Fig. 3. Compared with the main analysis, a higher frequency of dose reduction was recorded in the < 75-year group (*p* < 0.001), a finding consistent with the different starting doses of niraparib across matched groups; 23.1% of patients aged ≥ 75 years initiated treatment at 100 mg/day.

### Toxicity profile

All patients experienced at least one adverse events of any grade during niraparib treatment. Hematological toxicities were the most common grade ≥ 3 events, with variations between groups. Anemia was observed in 28.3% of patients in group A, compared to 3.8% in group B and 12.8% in group C. Similarly, neutropenia was reported in 19.4% of patients in group A, while it was not observed in group B and occurred in 15.4% of patients in group C. Thrombocytopenia showed a notable increase with age, affecting 24.2% of patients in group A, 15.4% in group B, and 33.3% in group C. Main non-hematological toxicities, such as gastrointestinal disorders or hypertension, were documented but were generally mild and limited to grade < 3. Dose reductions were required across all age groups, without a statistically significant difference (*p* = 0.08): patients in group A had the highest rate of dose reduction at 77.4% (n = 48), followed by 69.2% (n = 18) in group B, and 56.4% (n = 22) in group C. These reductions typically occurred early in treatment course, suggesting a critical period for managing toxicity and adjusting doses to improve tolerability.

Given the absence of dose reduction differences by age groups, we assessed clinical factors predicting dose reduction in the overall population. A multivariate analysis was performed to identify independent predictors, including age among the covariates, to explore potential associations with dose reduction. As reported in Table 3, the multivariable analysis demonstrated that patient underwent primary debulking surgery have a higher risk of dose reduction compared to interval debulking surgery (OR: 0.39, 95%CI: 0.18–0.85, *p* = 0.04). Platelet count, hemoglobin, and neutrophil count at the start of therapy, ECOG, and carboplatin dose showed no statistically significant impact.

**Table 3:** Multivariate analysis of predictive factors of dose reduction

Variables	____Multivariate Analysis____ OR (95% CI) *p*-value^a^	
**Age**		
Group A and Group B	0.55 (0.33–1.02)	0.35
Group C	1.00 ref	
**ECOG status**		
0	0.46 (0.27–1.32)	0.60
1	1.00 ref	
**Surgery**		
Primary cytoreductive surgery	1.00 ref	**0.04**
Interval cytoreductive surgery	0.39 (0.18–0.85)	
**Platelets count at starting niraparib **(10^3^/μL)	0.93 (0.85–1.02)	0.41
**Haemoglobin at starting niraparib **(g/dl)	0.99 (0.90–1.40)	0.78
**Neutrophils count at starting niraparib** (10^3^/μL)	0.98 (0.95–1.02)	0.44

Bold highlights statistically significant *p*-values

*ECOG* Eastern Cooperative Oncology Group,* CI* Confidence Interval,* OR *Odds Ratio,* ref *reference

^a^Calculated by logistic regression

**Fig. 3:**
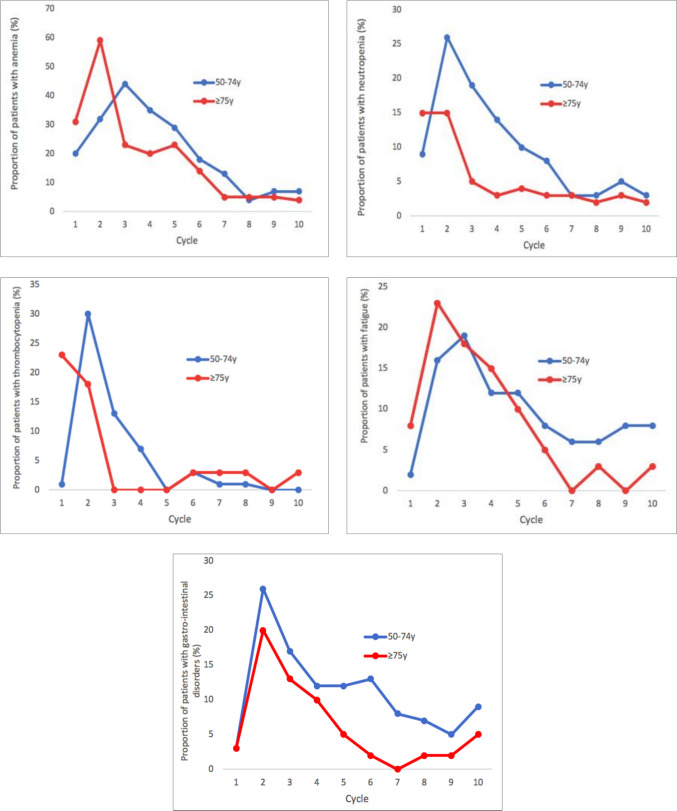
Treatment-emergent adverse events during niraparib maintenance in patients 50–74 years (groups A + B) versus ≥ 75 years (group C)

In the older cohort, approximately 17.9% (n = 7) of patients discontinued niraparib treatment prematurely (Table 2). The reasons for discontinuation included: 3 patients due to grade ≥ 3 hematological toxicity despite dose reduction, 2 patients due to worsening of pre-existing chronic renal insufficiency, 1 patient due to persistent fatigue, and 1 due to the development of acute myeloid leukemia, which subsequently led to death. Overall, 2 cases of myelodysplastic syndrome were observed: 1 patient in group A and 1 patient in group C. We also compared the toxicity profiles in terms of total adverse events of group C to those of younger patients (50–74 years), combining groups A and B. The figures reported in Fig. 3, showed that the trends of total adverse events (hematological, fatigue-related, and gastrointestinal toxicities) are largely comparable between the two age groups.

## Discussion

This retrospective real-world study analyzed the safety and efficacy of niraparib in a population of patients aged ≥ 75 years with advanced primary tubo-ovarian cancer. Our results showed that, in older population, niraparib was well tolerated, with a toxicity profile and oncological outcome comparable to younger cohorts. Despite the different baseline characteristics in the ≥ 75-year-old group as higher ECOG value, more frequent use of interval debulking surgery, and a proportion who did not undergo cytoreductive surgery, progression-free survival did not significantly differ from those observed in younger patients (*p* = 0.78). Moreover, dose reductions occurred less frequently in the elderly compared to younger patients, although this difference was not statistically significant (*p* = 0.08). Elderly patients exhibited a slightly higher incidence of grade ≥ 3 thrombocytopenia and no significant increase in non-hematologic or other severe adverse events was observed.

In the context of PARP inhibitors maintenance therapy, clinical trials have enrolled only a limited percentage of patients aged 65 and older: SOLO1 (13.8%), PAOLA-1 (36.2%), VELIA (39.7%), and PRIMA (39.4%) [2–4, 12]. This limited representation restricts the applicability of clinical trial data to real-world elderly populations. Recently, a meta-analysis by Maiorana et al. examined the efficacy of PARP inhibitors in elderly patients and concluded that age does not significantly affect the clinical benefits of PARP inhibitors [13]. This reinforces the rationale for the effective and routine use of PARP inhibitors in older populations.

Moreover, residual tumor following debulking surgery has been reported to be higher in elderly patients, representing the main prognostic factor for survival outcomes [14–18]. When providing suboptimal treatment to the elderly because of their age, numerous studies focused on primary treatment and demonstrated shorter survival outcome [19]. Warren et al., reported that only 37.6% of patients aged over 75 years underwent appropriate surgery, while Cloven et al. showed that optimal cytoreduction was achieved in only 29.5% of patients aged 60–79 years, decreasing further to 21.7% among those older than 80 years [17, 18]. Conversely, Joueidi et al*.* reported residual disease following cytoreduction surgery in 30% of patients aged ≥ 75 years, compared to 20% in those under 65 years (*p* = 0.04), with 70% of elderly patients achieving complete cytoreduction [16]. Similarly, in our cohort, a residual tumor > 1 cm or the inability to achieve optimal cytoreduction despite neoadjuvant chemotherapy was higher in group C (30.8%) compared to groups A and B, in which all patients achieved either no residual disease or a residual tumor ≤ 1 cm. Despite these challenges, the efficacy of niraparib was not compromised in our elderly population. This could suggest that advanced age should not preclude patients from receiving niraparib, as its therapeutic benefits remain consistent across age groups.

We noticed that severe thrombocytopenia was more frequent in group C (33.3%) compared to groups A and B (24.2% and 15.4%, respectively). This finding may be attributed to the more frequent use of medications such as anticoagulants, antiplatelet agents or to nutritional deficiencies (e.g., vitamin B12, folate) in the elderly population [20–23]. This is consistent with the ≥ 75 years subgroup of the PRIMA trial, which reported a severe thrombocytopenia rate of 35.3% in patients treated with the individualized starting dose, while the rate remained significantly higher (62.2%) in those who received the fixed standard dose [2].

Conversely, we found that the older population had a lower risk of grade 3–4 anemia (12.8%) compared to group A (28.3%). Our finding is also supported by the meta-analysis by Maiorana et al., which reported that older patients seem to have a lower risk of developing severe anemia [13].

Nonetheless, it should be underlined that 23.1% of patients in our elderly cohort started with an initial dose of 100 mg/day compared to only 1.1% of patients under 75 years.

A higher rate of dose reduction was observed in groups A and B (77.4% and 69.2%, respectively) compared to group C (56.4%); however, this difference was not statistically significant (*p* = 0.08). This slight difference may be attributable to the more frequent use of individualized starting doses among patients aged ≥ 75 years (100 mg/day in 23.1% of the older group vs 1.1% in the younger groups), as well as to the consequently different starting doses of niraparib across these groups (higher in groups A and B) (Table 2). When the analysis was restricted through propensity score matching to 39 patients per age group (< 75 and ≥ 75 years), the difference in dose reduction between the matched groups became statistically significant (*p* < 0.001), supporting the influence of the starting dose on subsequent dose reductions.

However, it should also be emphasized that, even when beginning with lower doses, older patients remain more susceptible to thrombocytopenia than to anemia. This suggests that the drug's toxicity is only partly dependent on dosage; it is also related to age and the characteristics of individual patients.

Interestingly, the dose reduction rate observed in elderly patients in our series was lower than the 61.5% reported in the PRIMA trial [2]. This difference is probably due to the use of the individualized starting dose and a more tailored dosing strategy in the elderly group, which considered general clinical conditions, comorbidities, residual post-chemotherapy toxicity, and age.

No substantial differences were observed in our cohort between the elderly and younger populations regarding non-severe hematologic adverse events and the incidence of severe neutropenia (Fig. 3). The overlapping trends observed in the toxicity curves reinforce the hypothesis that age alone does not substantially impact the overall safety profile of niraparib. These finding are consistent with data reported in previous clinical trials [2, 8].

No statistically significant differences were observed between groups in terms of dose discontinuation (*p* = 0.12). Moreover, the percentage of patients aged ≥ 75 years who discontinued niraparib due to grade ≥ 3 adverse events (17.9%) was comparable to the rate reported in the same population in the PRIMA trial (18.5%) [14]. This suggests that, with appropriate clinical management, the majority of older patients could continue therapy without significant interruptions. However, the occurrence of rare but severe events such as myelodysplastic syndrome and acute myeloid leukemia emphasizes the importance of continuous clinical monitoring, particularly in older patients who may already have compromised hematologic reserves.

Our data also suggest that older patients have comparable short-term survival outcomes to younger patients (HR 0.86, 95%CI: 0.45–2.41; *p* = 0.37), indicating that chronological age alone is not an independent predictive factor in tube-ovarian cancer. This is further supported by findings from the sub-analysis of IMPRESSS study, which enrolled 721 patients with epithelial ovarian cancer, including 226 (31%) patients aged ≥ 75 years. In this cohort, older patients who received treatment similar to that of younger women demonstrated similar survival rates. Notably, there was no association between age at diagnosis and progression free survival (*p* = 0.40), instead outcomes appeared to be primarily influenced by the type of treatment received [24].

We recognize that the retrospective design of the study and the lack of available quality of life data represent limitations in the interpretation of these findings. Additionally, the relatively small sample size may limit statistical power, and differences in baseline characteristics across the groups may have introduced potential confounding. Our findings should be interpreted considering the significant baseline differences observed between the ≥ 75-year group and the younger cohorts, particularly in ECOG performance status, surgical approach, and residual disease status. These imbalances may have introduced residual confounding in the progression-free survival analysis, independent of age or treatment-related variables. While propensity score-based methods are recommended to mitigate such biases, the relatively small sample size in our elderly subgroup would have resulted in substantial loss of statistical power and reduced interpretability of matched analyses. Consequently, our results should be interpreted as hypothesis-generating and confirmatory analyses using larger multi-center datasets with propensity-based adjustments are warranted.

Nonetheless, this is the largest real-world study investigating the management of very elderly patients treated with PARP inhibitors. This underscores the importance of tailoring treatment strategies in this population, including the individualized starting doses of niraparib.

As life expectancy continues to rise, the incidence of tubo-ovarian cancer in the elderly is expected to increase substantially. Consequently, oncologic management in this population is emerging as a critical issue for clinicians. Real-world data often provide a more accurate representation of older patients with various comorbidities and highlight the relevance of dose modifications and proactive management of adverse events to optimize treatment outcomes. These patients, particularly those aged ≥ 75 years, are often underrepresented in clinical trials, although the median age at tubo-ovarian cancer diagnosis is 63 years [1]. Our results reinforce the concept that age alone should not be considered a limiting factor when prescribing niraparib. With individualized starting doses and proactive monitoring strategies, older patients can benefit from maintenance therapy with manageable toxicity. These findings suggest that clinicians should consider incorporating elderly patients into maintenance regimens more systematically, avoiding age-based exclusions. Furthermore, future clinical trials should strive to include a representative proportion of older patients to enhance both external validity and equity in cancer care. Additional prospective studies, ideally including geriatric assessment tools, are warranted to refine treatment selection and enhance outcomes in this vulnerable population.

## Supplementary Information

Below is the link to the electronic supplementary material.

Supplementary file1 (DOCX 121 KB)

## Data Availability

In accordance with the journal’s guidelines, we will provide our data for independent analysis by a selected team by the editorial team for the purposes of additional data analysis or for the reproducibility of this study in other centers if such is requested.
