# Supplementary material for: Real-life observational study on niraparib in older patients with primary tubo-ovarian cancer: a focus on safety and efficacy
Source: Int J Clin Oncol. 2025 Nov 4;30(12):2652–61. doi: 10.1007/s10147-025-02914-y (PMC12644187; doi:10.1007/s10147-025-02914-y)
Supplement: Supplementary file 1 — Supplementary file1 (DOCX 121 KB) [file 10147_2025_2914_MOESM1_ESM.docx]

**Real-life observational study on niraparib in older patients with primary tubo-ovarian cancer: a focus on safety and efficacy**

Adriana Ionelia Apostol^1#^, Matteo Bruno^1#^*, Carolina Maria Sassu^1^, Serena Maria Boccia^1^, Laura Vertechy^1^, Giorgia Russo^1^, Ilary Ruscito^1^, Filippo Maria Capomacchia^1^, Giovanni Scambia^†1,2^, Anna Fagotti^1,2^, Claudia Marchetti^1,2^

^1^Dipartimento Scienze della Salute della Donna, del Bambino e di Sanità Pubblica, Fondazione Policlinico Universitario Agostino Gemelli, IRCCS, Rome, Italy.

^2^Dipartimento Scienze della Vita e Sanità Pubblica, Università Cattolica del Sacro Cuore, Rome, Italy.

# These authors contributed equally to this work.

***Corresponding Author**: Matteo Bruno, MD

Dipartimento Scienze della Salute della Donna, del Bambino e di Sanità Pubblica, Fondazione Policlinico Universitario Agostino Gemelli, IRCCS, via Largo Francesco Vito 1, Rome, Italy.

E-mail: brunomatteo2@gmail.com

Tel.: + 39-0630153733

orcid.org/0000-0003-4372-4220

**Supplementary Figure 1** Distribution of maintenance therapy types in patients aged ≥75 years with primary high-grade serous tubo-ovarian carcinoma

**1** rucaparib

**21** olaparib

**39** niraparib

**4** bevacizumab + olaparib

**61** Parp-inhibitors

monotherapy

**23** bevacizuamb

**88** patients received maintenance therapy:

**58** patients did not receive maintenance therapy due to:

- **34**: clinical conditions;

- **24**: platinum-refractory disease;

**146** patients treated at our center

**137** patients excluded: therapy continued at other hospital

**283** patients aged ≥75 years,

diagnosed with primary high-grade serous tubo-ovarian carcinoma between 2019 and 2023 at Fondazione Policlinico Agostino Gemelli, IRCCS, Rome, Italy

**Supplementary Figure 2** Progression-free survival across the three groups: A (50-64 years), B (65-74 years), and C (≥75 years, excluding those who never underwent cytoreduction surgery)

**Supplementary Figure 3** Progression-free survival among patients included in the propensity score-matched groups (<75 vs ≥75 years)


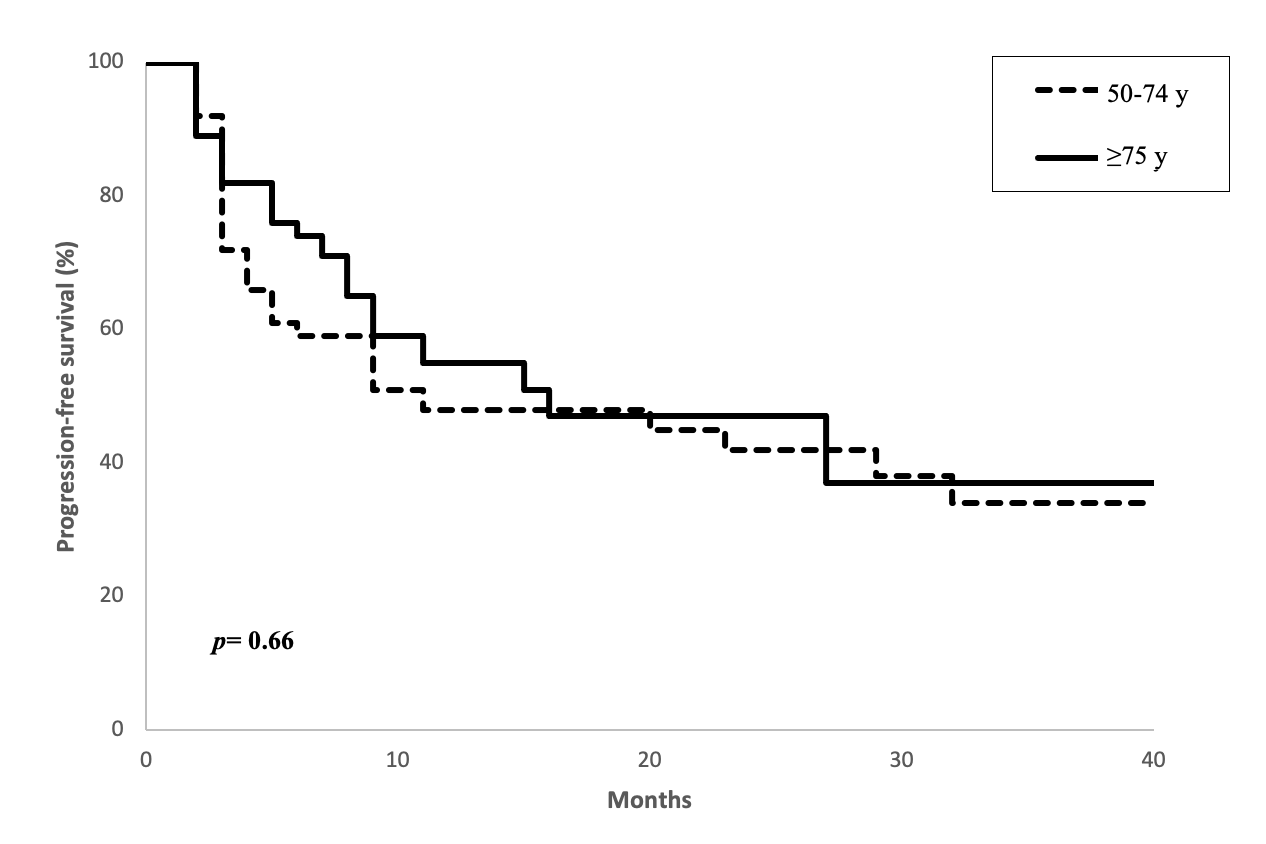


**Supplementary Table 1** Demographic and clinical characteristics of patients included in the propensity score-matched groups

| **Variables** | **Age <75 years**  **(n= 39)** | **Age ≥ 75 years**  **(n =39)** | ***p*-value** |
| --- | --- | --- | --- |
| **Age at diagnosis - median (SD)** | 61.6 (± 7.3) | 78.8 (± 3.9) | <0.0001 |
| **HRD Status, n (%)** |  |  | 0.48 |
| HR proficient | 11 (28.2%) | 4 (10.3 %) |  |
| HR deficient | 6 (15.4%) | 4 (10.3%) |  |
| Unknown^1^ | 22 (56.4%) | 31 (79.4%) |  |
| ***BRCA* status, n (%)** |  |  | 0.50 |
| *BRCA1/2* wild-type | 39 (100%) | 38 (97.4%) |  |
| *BRCA1/2* mutated | 0 | 1 (2.6%) |  |
| **Histological subtype, n (%)** |  |  | 1.00 |
| Serous | 39 (100%) | 39 (100%) |  |
| Others | 0 | 0 |  |
| **FIGO stage at diagnosis, n (%)** |  |  | 0.55 |
| IIIA | 1 (2.6%) | 1 (2.6%) |  |
| IIIB | 1 (2.6%) | 2 (5.1%) |  |
| IIIC | 20 (51.3%) | 21 (53.8%) |  |
| IVA | 8 (20.5%) | 3 (7.7%) |  |
| IVB | 9 (23.0%) | 12 (30.8%) |  |
| **Surgery, n (%)** |  |  | 0.001 |
| Primary debulking surgery | 8 (20.5%) | 12 (30.8%) |  |
| Interval debulking surgery | 31 (79.5%) | 18 (46.2%) |  |
| No cytoreduction surgery | 0 | 9 (23.1%) |  |
| **Residual tumor, n (%)^2^** |  |  | 0.13 |
| Residual tumor = 0 | 38 (97.4%) | 26 (86.7%) |  |
| Residual tumor ≤ 1 cm | 1 (2.6%) | 1 (3.3%) |  |
| Residual tumor >1 cm | 0 | 3 (10%) |  |
| **Niraparib starting age- median (SD) (years)** | 62.3 (± 7.2) | 79 (± 3.7) | <0.0001 |
| **Niraparib starting weight - median (SD) (kg)** | 60.5 (± 9.5) | 61.8 (± 8.6) | 0.54 |
| **ECOG, n (%)** |  |  |  |
| 0 | 35 (89.7%) | 17 (43.6%) |  |
| 1 | 4 (10.3%) | 22 (56.4%) |  |
| **Niraparib starting dose, n (%)** |  |  | 0.003 |
| 300 mg/die | 2 (5.1%) | 0 |  |
| 200 mg/die | 37 (94.9%) | 30 (76.9%) |  |
| 100 mg/die | 0 | 9 (23.1%) |  |
| **Niraparib dose reduction, n (%)** |  |  | <0.001 |
| Yes | 32 (82.1%) | 22 (56.4%) |  |
| No | 7 (17.9%) | 17 (43.6%) |  |
| **Niraparib duration - median (SD) (months)** | 9 (± 9.8) | 8.8 (± 10.2) | 0.11 |

Abbreviations: SD, standard deviation; FIGO, International Federation of Gynecology and Obstetrics; HRD, homologous recombination deficiency; ECOG, Eastern Cooperative Oncology Group.

^1^HRD status was available for few patients due to the time interval considered (2019-2023).

^2^Patients aged ≥75 years without cytoreductive surgery (n= 9) were excluded from the residual tumor analysis.
